# Macrodystrophia lipomatosa: four case reports

**DOI:** 10.1186/1824-7288-36-69

**Published:** 2010-10-22

**Authors:** Rizwan A Khan, Shagufta Wahab, Ibne Ahmad, Rajendra S Chana

**Affiliations:** 1 Division of Paediatric Surgery, JNMCH, AMU, Aligarh, India; 2 Dept of Radiodiagnosis, JNMCH, AMU, Aligarh, India

## Abstract

**Aim:**

Macrodystrophia lipomatosa is a rare cause of gigantism of limb which can be confused with other common causes like congenital lymphedema. It presents usually with loss of function and cosmetic problems. Four cases are described with emphasis on clinical presentation, differential diagnoses, imaging and treatment options.

**Methods & Results:**

Four patients of macrodystrophia lipomatosa were thoroughly examined and subjected to investigations.

**Conclusion:**

Besides diligent clinical examination, imaging and histopathology are crucial in clinching the diagnosis.

## Introduction

Macrodystrophia lipomatosa, hamartomatous enlargement of the soft tissue components leading to localized or generalized gigantism of a limb, is a rare congenital disorder which can present anywhere from infancy to late adulthood. There are various causes which can lead to increased size of one or several fingers or toes. Presentation in pediatric age group leads to considerable confusion and till such time when the diagnosis is reached, various descriptive terms are given to such an anomaly [1]. A variety of terms have been used to nominate the condition like macrodactyly, megalodactyly, digital gigantism, macromelia, partial acromegaly, macrosomy, and limited gigantism [1-8].

We present here four cases of the anomaly with the aim to high lighten the clinical features, differential diagnoses and the treatment protocol to be followed in these patients.

### Case no.1

A 5-year-old girl had a history of steady enlargement of the right-lower limb since birth and mainly the medial three toes of the right foot. There was loss of function but there was no history suggestive of trauma, pain, skin changes or family history. The plain radiographs revealed perceptible increase in the soft tissues elements of the foot. The medial three short bones of the foot appeared a little bulkier but the cortex and their normal form was preserved. Biopsy demonstrated profuse amount of fatty tissue with proliferation of subcutaneous nerves (Table 1).

**Table 1:** Showing the various presentation, involvement and histopathology of the patients

Patient	Sex/Age	Affected Region	Clinical Features	Histopathological Features
1	5y/F	Medial aspect of right foot	Increased size of right foot with inability to walk and sit	Increased fatty tissue with proliferation of subcutaneous nerves
2	7y/F	Medial aspect of right foot	Decreased mobility, recurrent injury	Lipomatosis infiltrating nerves and mild increase in the fibrous tissue
3	6y/M	Medial aspect of left foot	Difficulty in playing football, problems in putting on his footwear.	Encapsulated lobules of well differentiated fatty tissue.
4	2y/M	Medial aspect of left foot	Gradual increase in the size of left foot	Increase in the fatty component of the subcutaneous tissue.

### Case no.2

**Figure 1:**
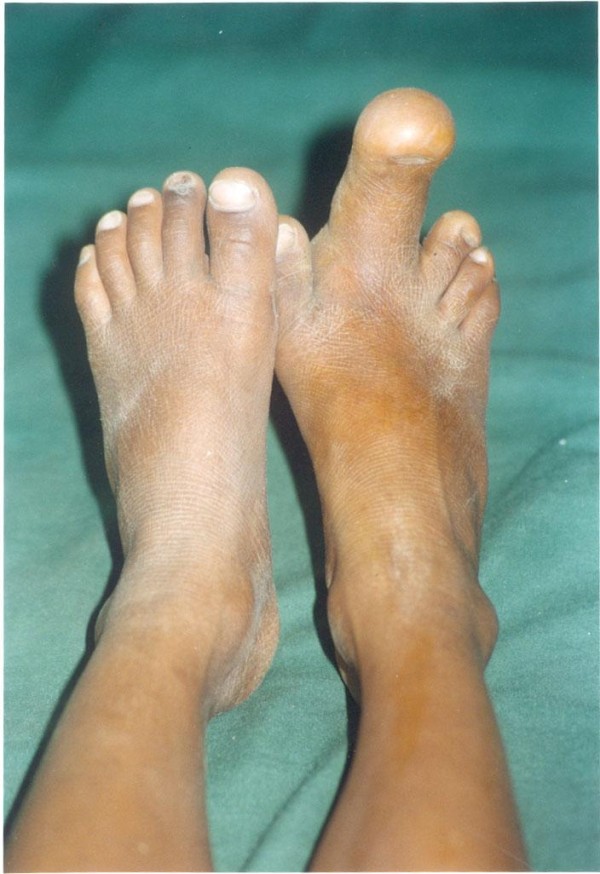
**Clinical image showing grotesque bulbous enlargement of 2^nd ^digit of the right foot**.

**Figure 2:**
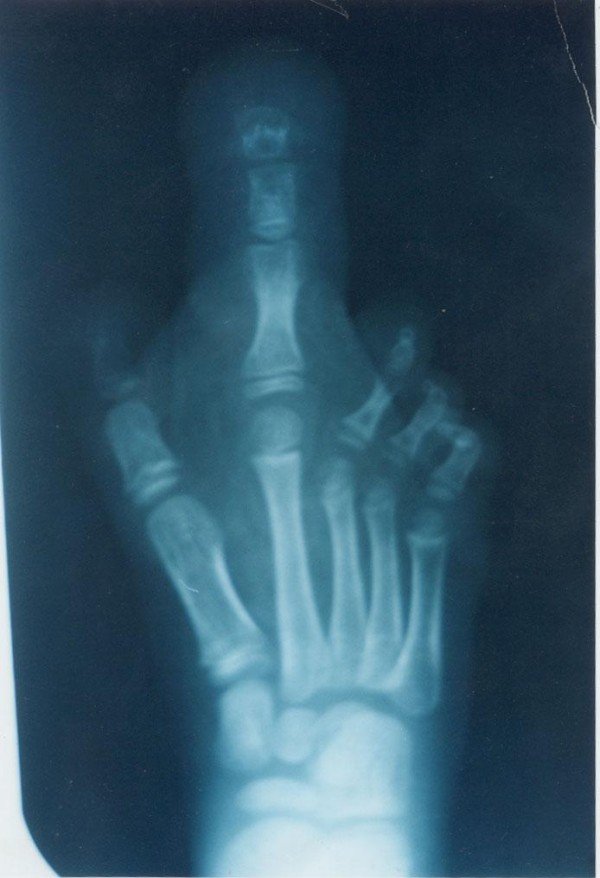
**X-ray image showing hypertrophy of the soft tissues of the 2^nd ^digit of right foot. The phalanges of the involved digit also appear enlarged**.

A 7-year-old girl was referred with excessive growth of the 2^nd ^toe of the right foot. The 2^nd ^toe of the right foot was unusually large with an increase in subcutaneous tissue which was visibly more prominent on the ventral aspect (Figure 1). Except for the fact that she had difficulty in walking and recurrent injury to the foot, rest of her history and physical examination was non contributory. Plain radiography of the foot revealed enlarged metatarsals and phalanges of the 2^nd ^toe of the right foot (Figure 2). The soft tissue was also increased. The biopsy was suggestive of lipomatosis infiltrating nerves and mild increase in the fibrous tissue was also noted.

### Case no.3

**Figure 3:**
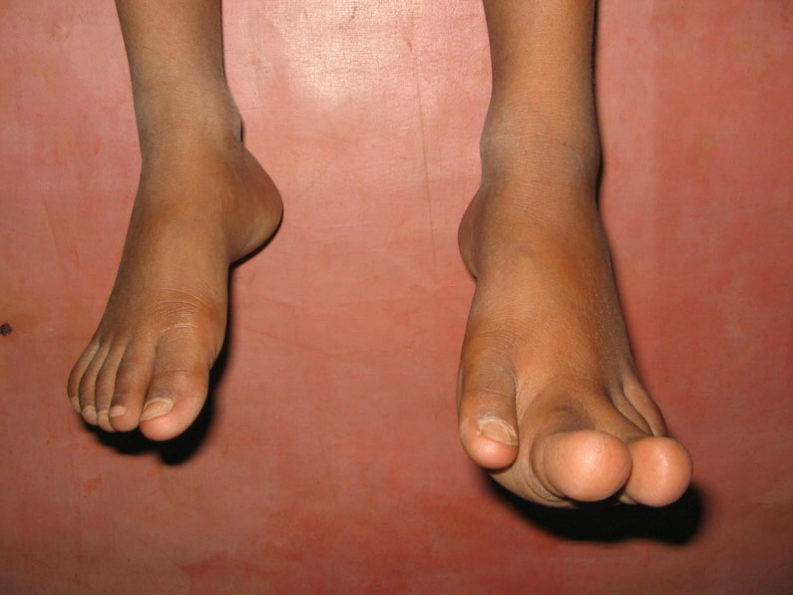
**Photograph showing enlarged 2^nd ^and 3^rd ^toes of the patient**.

**Figure 4:**
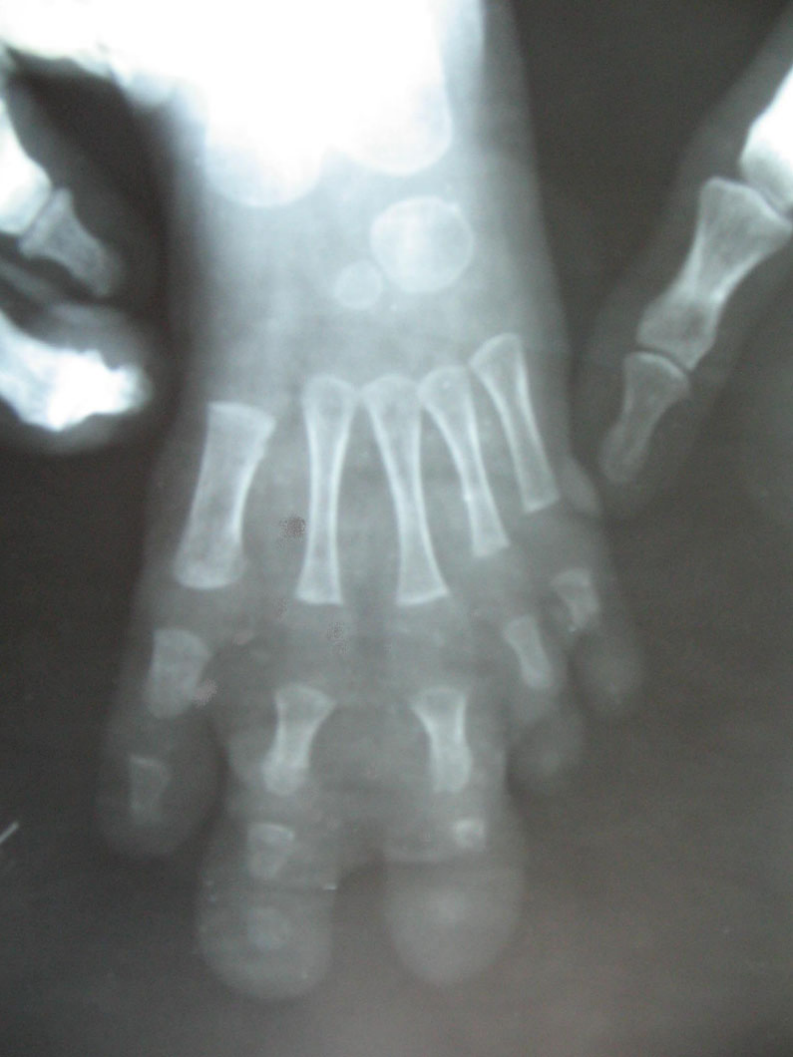
**Enlarged 2^nd ^and 3^rd ^toes with well appreciated increase in size of metatarsals, phalanges and soft tissues on x-ray**.

A 6-year-old boy presented with enlargement of his left foot. Parents noticed gradual enlargement of the left foot, mainly involving the second and third ray (Figure 3). Initially the boy had difficulty in playing football and later on had problems in putting on his footwear. On examination, there were no integumentary or neurological findings. However, there was enlargement of 2^nd ^and 3^rd ^toe with sparing of rest of the toes. X-rays revealed mainly the enlargement of the soft tissue part and some increase in size of phalanges (Figure 4). Biopsy revealed encapsulated lobules of well differentiated fatty tissue.

### Case no.4

A two-year-old boy presented with increased size of the toes of left foot which were gradually increasing since 1 year of age. There was no history of increase since birth. On examination we could find that the boy had unusual hypertrophy of the left lower extremity from distal leg onwards with predominant involvement of the 2^nd ^and 3^rd ^toes. There were some maculopapular lesions in the involved limb. The other lower limb was normal. Physical examination showed subcutaneous increase in the tissue that was not tender on palpation and there was no evidence of edema (patient was diagnosed as congenital lymphedema of the foot at an outside facility was under the treatment for the same). Assessment of the other systems showed no abnormality. Radiological examination showed increase in length and diameter of the metatarsals and toes with appreciable increase in the soft tissue component. Punch biopsy revealed increase in the fatty component of the subcutaneous tissue.

## Discussion

Macrodystrophia lipomatosa (ML) is a rare congenital anomaly characterized by an abnormal overgrowth of the mesenchymal elements resulting in gigantism of single or multiple digits or the entire limb. Sometimes it may have bilateral involvement of the limbs [2]. Although there have been only few anecdotal reports of involvement of entire limb and even the abdominal wall involvement has been reported [2,3], in our series two of the patients had involvement of entire limb. The age of presentation is in consistent and the clinical findings can be recognized as early as in the neonatal period to late adulthood. Although there is no gender predilection, slight male preponderance is seen. The overgrowth appears to develop in a specific sclerotome region of the body [3]. Generally, the lateral aspect of the upper limb (i.e. along the median nerve distribution) and the medial aspect of the lower limb (i.e. along the plantar nerve distribution) are affected. Distal limb involvement is seen predominantly. The lower limb is more often involved than the upper limb and the 2nd and 3rd digits are more commonly affected [3,4]. The affected region continues to grow only until puberty at which time it reaches a plateau.

Macrodystrophia Lipomatosa is a congenital anomaly but it is certainly not a hereditary disease [5]. Various hypotheses have been proposed regarding the etiopathogenesis of ML. These include lipomatous degeneration, fetal circulation abnormality, and damage of extremity bud and errors in the segmentation in intrauterine life and hypertrophy of the concerned nerve [5-8]. The gamut of nomenclature, as given by different authors, produces considerable confusion regarding the exact terminology and also difficulty in setting up the criteria for diagnosing the disease especially vis-à-vis fibrolipomatous hamartoma (FLH) [9]. Feriz in 1925 coined the term Macrodystrophia lipomatosa. Barsky in 1967 gave a detailed description of local gigantism and he differentiated two forms of it, namely static and progressive. The progressive form of the disease described by Barsky is analogous to what Feriz described [10].

Fibrolipomatous hamartoma (FLH) is another heterogeneous group of lesions which produces digital overgrowth and can be confused with ML. It usually presents as an isolated nerve lesion and associated with intramuscular fat deposition [11]. But in ML, besides fat deposition in nerve sheaths, subcutaneous and muscle compartment, there is involvement of periosteum also leading to the bony changes, such as hypertrophy, exostosis, ankylosis of interphalangeal joints and fatty invasion of the medullary cavity [10].

In ML, the most prominent histopathological finding is the increase in adipose tissue scattered in a fine lattice of fibrous tissue, which involves the bone marrow, periosteum, muscles, nerve sheaths, and subcutaneous tissues [12]. Neural enlargement and irregularity may be also be seen, most commonly involving the median nerve in the hand and the plantar nerves in the foot [12]. The periosteum can be seen studded with small nodules of chondroblasts, osteoblasts, and osteoclasts. These nodules become larger and more numerous toward the distal ends of the phalanges making them elongated, broadened, and splayed at the distal ends often called mushroom-like appearance [13]. There may be hypertrophy and degeneration in bone structure and increase in the medullary bone density. Trabecular structure of bones is characteristically well-preserved [13]. In adult patients, secondary osteoarthritic changes like joint space narrowing, subchondral cysts and large osteophytes can be seen [4,12].

The problems associated with a patient of ML can be twofold, cosmetic and mechanical. Since overgrowth mainly involves the volar aspect, it can produce dorsal deviation of affected parts. This may lead to interference in normal day to day activities or make patient prone to repeated injury as was seen in our patients. However, mechanical problems may develop like secondary osteoarthritis and compression of neurovascular structures causing impairment of function [14]. Cosmetic problem is the usual presenting complaint in all ages but mechanical problems are encountered in adolescence due to secondary degenerative joint changes causing reduced function. Osteophyte overgrowth may also cause compression of adjacent nerves and vessels, most commonly seen is carpal tunnel syndrome [14]. The other entrapment syndromes that can be are cubital and tarsal tunnel syndromes [15]. Electroneurography and nerve conduction velocity tests performed in these patients may reveal slowed distal motor and sensory conduction, local (segmental) conduction block or slowing of the peripheral nerves at the entrapment sites [15]. Other associated problem seen are lipomatous growths in intestines and in other tissues, calvarial abnormalities, pigmented nevus, pulmonary cysts syndactyly, polydactyly, clinodactyly, brachydactly and symphalangism may be seen in ML [16]. Bilateral involvement is very rare. Also there is no familial predominance or neurocutaneous involvement (as against the neurofibromatosis) [18]. There is a known association of hemihypertrophy with conditions such as the Beckwith-Wiedemann syndrome with its underlying tumors, especially Wilms' tumor (abdominal sonography is mandatory as was performed in all of our cases) [17,18].

Typical X ray findings of ML includes excessive growth of soft tissue as well as osseous tissue, presence of radiolucent areas due to presence of adipose tissue and degenerative joint disease. Excessive growth of the bone within the area innervated by nerve and fat tissue proliferation within muscle fibers are the characteristic findings detected on CT scan. The volar aspect of the fingers is disproportionately involved [12,13]. Widening at the distal end of the bones gives the characterstic mushroom-like appearance [12,13]. The excessive fat seen in ML is not encapsulated and MRI can easily demonstrate the fatty infiltration of the muscles. There may be linear hypointense fibrous bands noted within this abnormal fat. There is osseous hypertrophy and cortical thickening in the affected part of the body and this may lead to exostoses like bony outgrowths from the involved bone. MR imaging also reveals a redundancy of fatty tissue and fibrous thickening of a nerve [6].

There are certain conditions which can present with localized limb hypertrophy. The differential diagnosis of ML includes neurofibromatosis type 1 (plexiform neurofibroma), fibrolipomatous hamartoma (FLH), lymphangiomatosis, hemangiomatosis and Klippel-Trenaunay-Weber syndrome, Mafucci syndrome, Ollier disease and Proteus syndrome. But in all of these differential diagnoses there is a positive family history and are characterized by cutaneous or systemic manifestations [16]. For example in Neurofibromatosis, besides positive family history, there are caf'e-au-lait spots on the skin, soft tissue nodules and it is usually bilateral and involvement of distal phalanx is not that severe. Klippel-Trenaunay-Weber syndrome is characterized by the presence of cutaneous hemangiomas and varicose veins. Neither lymphangiomatosis nor hemangiomatosis show osseous growth. Patients of Proteus syndrome typically present with skull abnormalities, pigmented naevi, lung cysts, dermatologic changes like palmar and plantar cerebroid thickening and intra-abdominal lipomas [17].

Usually the intervention is usually carried out due to cosmetic reasons, but the guiding surgical principle in managing this condition should be improving cosmetic appearance as well as preserving neurologic function [16]. Surgical interference is indicated depending upon patient's symptoms, age, and extent and severity of the disease. For lesion involving median and plantar region, conservative approach has been advocated [16,18]. We should also keep in mind that digital enlargement associated with ML usually stops at puberty. In a localized form of disease, removal of a ray is the most appropriate procedure that can lead to a cosmetic improvement and does not cause any functional or neurologic problems as well. Multiple debulking procedures, epiphysiodesis, and various osteotomies are indicated for more severe form of the disease. Epiphysiodesis is not an effective method when performed alone as it may lead to loss of function. These procedures should be planned and used judiciously so as to achieve the best possible outcome. Some authors have advocated use of preoperative angiography to help improve the management [10,16,18]. Thus, the surgical treatment is quite a challenge because, despite the removal of adequate tissue, the difference in size may not be very apparent. Besides, the incidence of nerve injury following extensive debulking or lesion removal is approximately 30%-50%. Another upsetting fact is the high recurrence rate which to the tune of 33%-60% [9,18].

## Conclusion

ML is progressive hamartomatous enlargement of the fibrofatty tissue involving all the layers of soft tissue and even bone more commonly leading to localized gigantism Diagnosis is accomplished on the basis of clinical and radiological evaluation which can be confirmed on histopathological examination The management is mainly surgical but the outcome may not be very gratifying.

## Consent Section

Written informed consent was obtained from the patient for publication of this case report and accompanying images. A copy of the written consent is available for review by the Editor-in-Chief of this journal

## Competing interests

The authors declare that they have no competing interests.

## Authors' contributions

RAK conceived the study and the design. SW conceived, participated in design and coordinated the study. SW involved in correspondence as well. IA and RSC were involved acquisition, analysis and interpretation of data. All authors read and approved the final manuscript.
